# 

**Published:** 1875-05

**Authors:** 


					﻿CONTENTS:
Original Communications:
Art. I. Various Uses of Chloride of
Zinc. By J. E. Nichols, M.D.......321
Art. II. Koumiss as a Remedial Agent
—Report of Cases. By R. L Leonard,
M.D..............................326
Progress in Medical Sciences :
Art. I. Progress of Obstetrics. By E.
Warren Sawyer, M.D...............330
Art. II. Progress of Gynæcology. By
A. Reeves Jackson, M.D...........334
Selections.
Impressions of American Surgery....340
Case of Aneurism of the Abdominal
Aorta............................353
A Strychnia Eater, etc............354
Spinal Arthropathies..............355
Similarity between Red Blood-Corpus-
cles of Man and those of certain other
Mammals, etc.....................376
Explanatory Note in Regard to Diagno-
sis of Blood Stains..............390
Reports of Societies.................368
; Editors’ Book Table.................393
Mortality Statistics, Chicago........398
Illinois State Medical Society.
The Twenty-fifth Annual Session will be held in the city of Jacksonville,
on the third Tuesday in May (18th), 1875, at 9 o’clock, a. m. The following
Committees are expected to report :
Committee on Practical Medicine — E. P. Cook, M. D., Mendota, Chair-
man.
Committee on Surgery — Edwin Powell, M.D., Chicago, Chairman.
Committee on Obstetrics — Hiram Nance, M.D., Kewanee, Chairman.
Committee on Drugs and Medicines — Wm. E. Quine, M. D., Chicago,
Chairman.
Committee on Necrology — E. Ingals, M.D., Chicago, Chairman.
Committee on Opthalmology — E. L. Holmes, M.D., Chicago, Chairman.
Committee on Otology — S. J. Jones, M.D., Chicago, Chairman.
•	SPECIAL COMMITTEES.
Committee on Diseases of Children—J. O. Hamilton, M.D., Jerseyville,
Chairman.
Committee on Diseases of Respirat >ry Organs — H. A. Johnson, M.D.,
Chicago, Chairman.
Committee on Galvano-Therapeutics—David Prince, M.D., Jackson-
ville, Chairman.
Committee on Diseases of Women — J. T. Pearman, M.D., Champaign,
CAowwb.
Committee on Diseases of the Nervous System — J. S. Whitmire, M.D.,
Metamora, Chairman.
Committee on Medical Jurisprudence—R. J. Patterson, M.D., Batavia,
Chairman.
Committee on Dermatology — Chas. G. Smith, M.D., Chicago, Chairman.
Committee on Public Address — J. H. Hollister, M.D., Chicago, Ch'n.
Each local Society shall have the privilege of sending to the Society one
delegate for every five of its regular resident members, and one for every
additional fraction of more than half of this number. The faculty of every
regularly constituted medical college or chartered school of medicine, shall
have the privilege of sending two delegates. The professional staff of
every chartered or municipal hospital, and every other permanently organ-
ized medical institution of good standing, shall have the privilege of send-
ing one delegate.
The Members by Invitation shall consist of practitioners of reputable stand-
ing from any part of the United States. They shall receive their appoint-
ment by invitation of the meeting, after an introduction from any of the
members present, or from any of the absent permanent members. They shall
hold their connection with the Society until the close of the session at which
they were received, and may participate in the discussions without the
right of voting.
The Permanent Members shall consist of regular graduates from reputable
schools of medicine, who have served in the capacity of delegates, or such
other physicians as may be proposed by two members of this Society, re-
ported on favorably by the Committee of Investigation, and receiving a
two-thirds vote of all the members present.
A circular will be sent to each member, from the Committee of Arrange-
ments, giving all necessary information relative to the programme and the
arrangements made with the different railroads in regard to reduction of
fare, etc.
. T. Davis Fitch, M.D., Permanent Secretary,
296 W. Mcnroe St., Chicago.
				

